# Supplementary material for: IL17RB and IL17REL Expression Are Associated with Improved Prognosis in HPV-Infected Head and Neck Squamous Cell Carcinomas
Source: Pathogens. 2023 Apr 7;12(4):572. doi: 10.3390/pathogens12040572 (PMC10143491; doi:10.3390/pathogens12040572)
Supplement: Supplementary file 1 [file pathogens-12-00572-s001.zip › pathogens-2143972-supplementary/Supplementary files.pdf]

Supplementary Figure S1

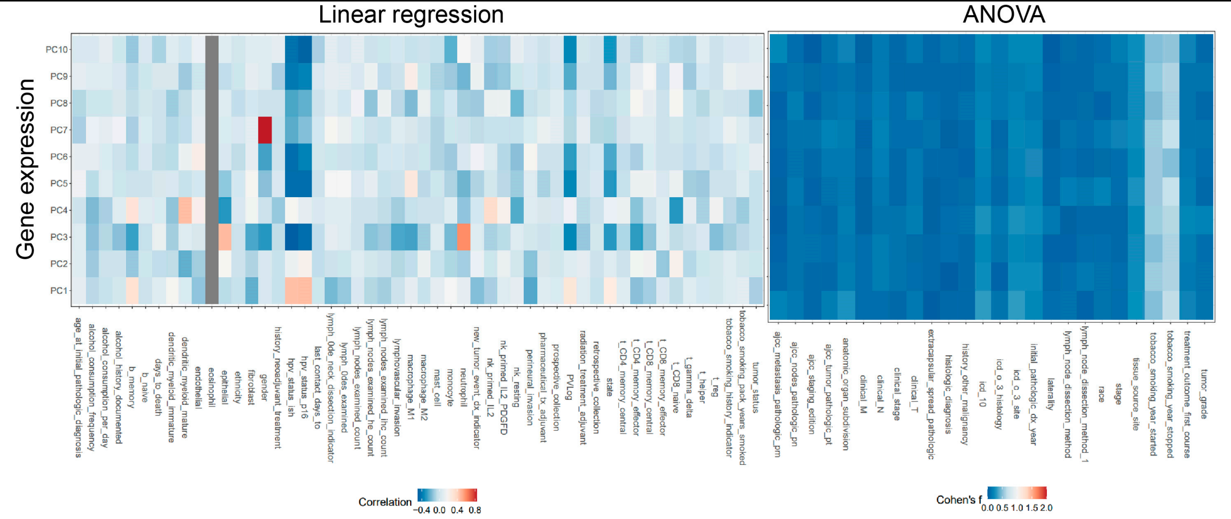

Supplementary Figure 1. The correlation between principal components (PCs) of gene expression and all covariates. As for numerical covariates (i.e. cell fraction, total living days), linear regression was performed while ANOVA was applied to categorical covariates (i.e. anatomic organ subdivision). Neutrophil, epithelial, dendritic cell infiltration, gender and HPV infection were found to associated with gene expression of TCGA-HNSC patients. However, in this study, we only focused on the single effector HPV infection.

Supplementary Figure S2

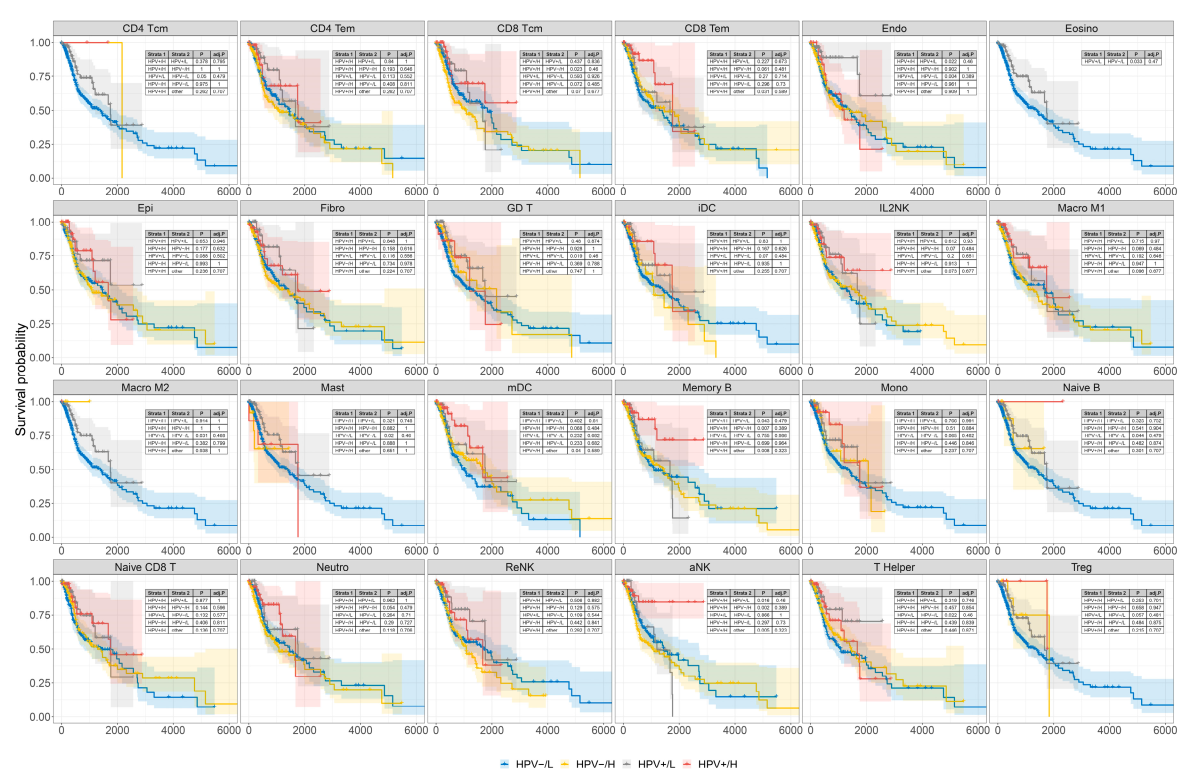

Supplementary Figure 2. The KM survival curves (y-axis, survival probability; x-axis, days) constructed for combinations of HPV infection and 24 different cell TS expression in HNSC patient tumors. Each cell TS expression was split by the median into L and H groups. HNSC patients with

both HPV infection and high expression of either memory B cell TS or aNK TS had significantly improved prognosis compared to other groups.

Supplementary Figure S3

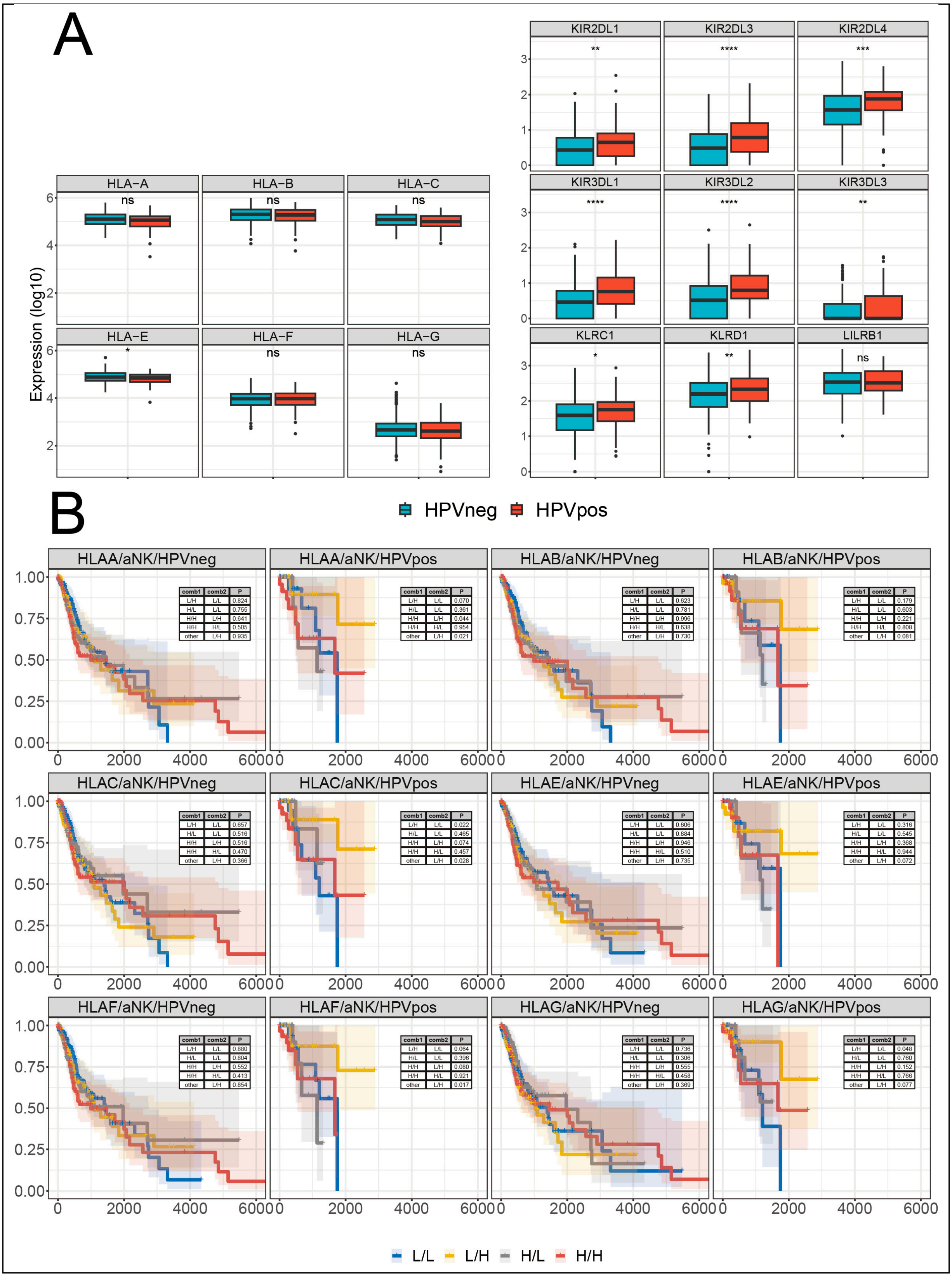

Supplementary Figure 3. Abundance and survival analysis of MHC-I molecules and cognate NK cell receptors. **(A)** Box plots comparing MHC-I variants and NK cell MHC-I cognate inhibitory receptor gene expression between HPV negative and positive HNSC patients. *HLA-E* were significantly downregulated while *KIR2DL1, KIR2DL3, KIR2DL4, KIR3DL1, KIR3DL2, KIR3DL3, KLRC1 and KLRD1* were all significantly upregulated in HPV infected HNSC patients. **(B)** Combined HNSC patient survival analysis stratified for MHC-I variants and aNK TS expression in both HPV negative and positive patients. KM curves (y-axis, survival probability; x-axis, days) display HPV-infected or free HSNC patient survival plotted in all four combinations for each stratum (L/L, L/H, H/L, and H/H, both L and H groups were split by the median gene or cell TS expression). For patients with HPV infection, only low expression of MHC-I molecules and high expression of aNK TS resulted in enhanced prognosis.

Supplementary Table S4

Supplementary File 4. All differentially expressed genes (logFC >= 1.5 or logFC <= -1.5 and FDR <= 0.05) in HPV infected HNSC patients.

Supplementary Figure S5

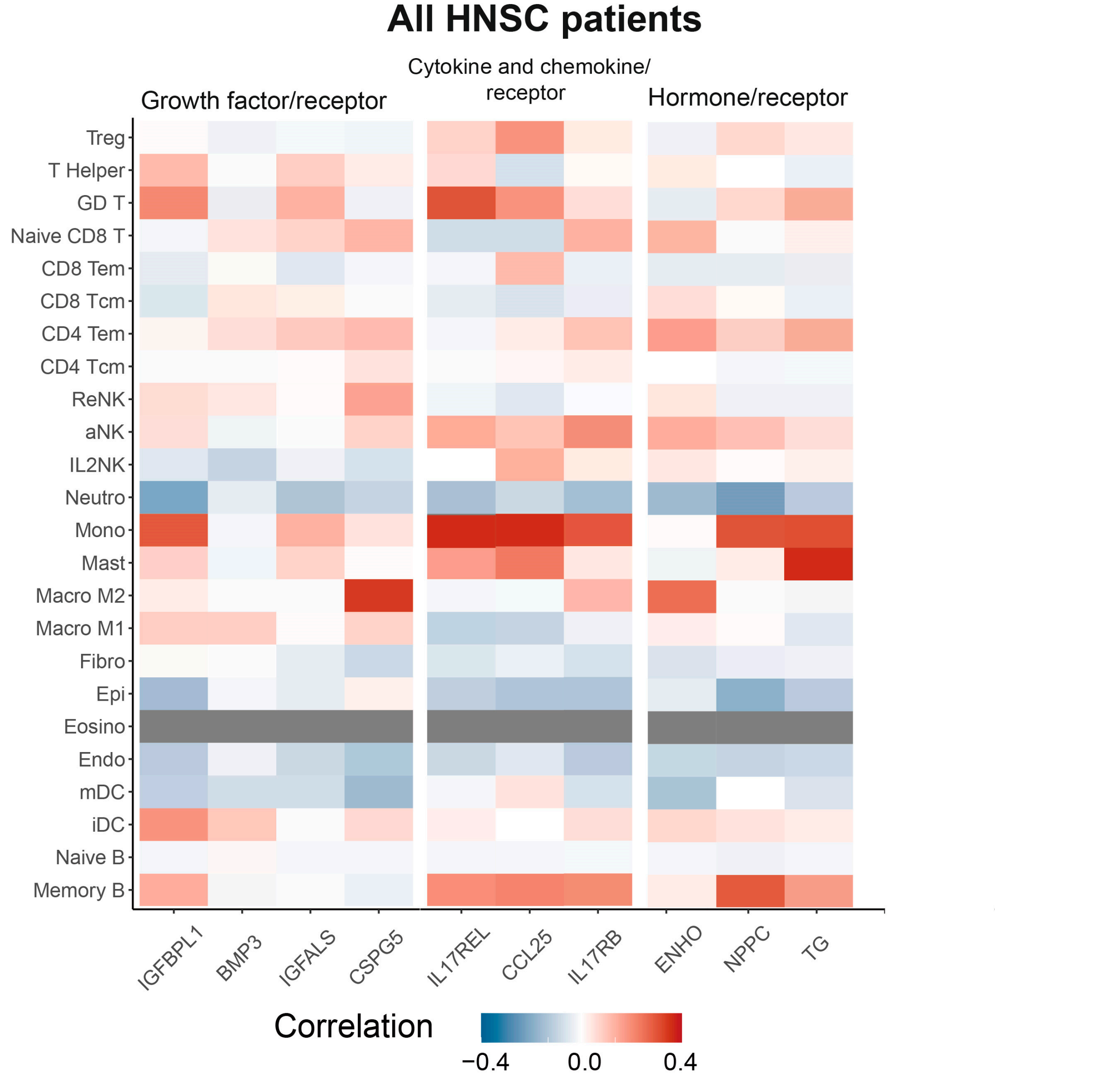

Supplementary Figure 5. The correlation heatmap of significantly upregulated secretome genes and all cell TS in all HNSC patients (both HPV infected and HPV uninfected patients).

Supplementary Figure S6

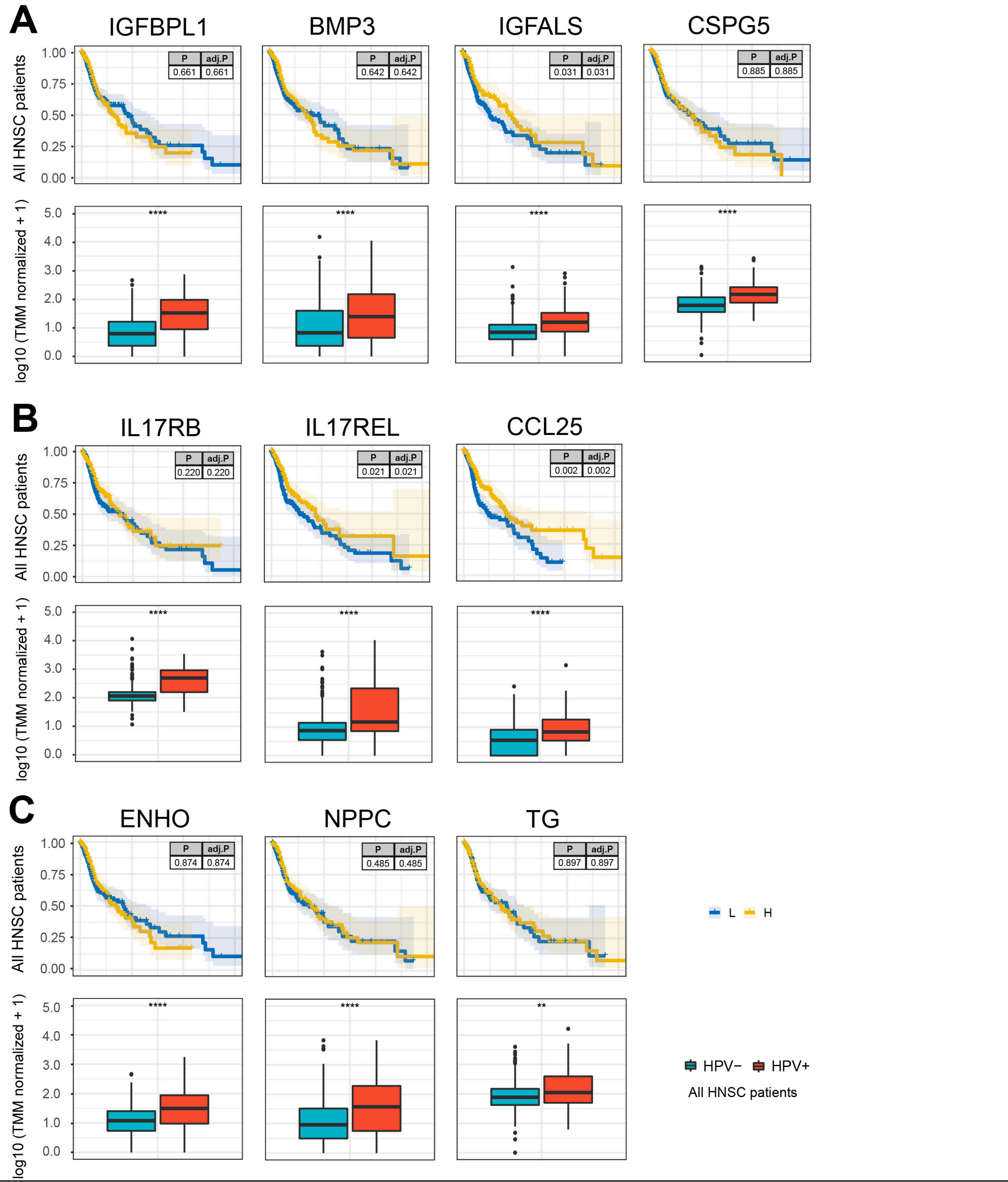

Supplementary Figure 6. Survival analysis and box plots of selected secretome genes in all HNSC patients. **(A)** KM curves (y-axis, survival probability; x-axis, days) and box plots constructed for growth factor, **(B)** cytokine and chemokine, **(C)** hormone and relevant receptor genes that were upregulated in all TCGA-HNSC patients. Each gene expression was split by the median into L and H groups. The box plots were grouped by HPV- and HPV+ HNSC patients with statistical tests. All eleven secret protein genes were upregulated in HPV infected HNSC patients.

Supplementary Figure S7

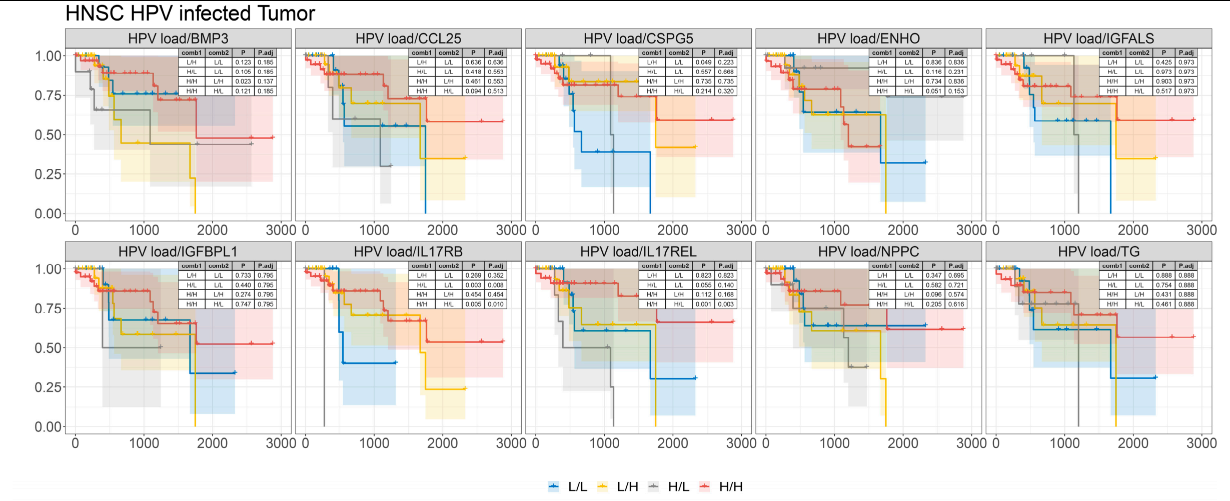

Supplementary Figure 7. Combined HNSC patient survival analysis stratified for HPV viral loads and all the upregulated secret protein genes. KM curves (y-axis, survival probability; x-axis, days) display HPV-infected HSNC patient survival plotted in all four combinations for each stratum (L/L, L/H, H/L, and H/H, both L and H groups were split by the median viral load or gene expression). For patients with higher HPV viral loads, only high expression of either *IL17RB* or *IL17REL* resulted in enhanced prognosis.

Supplementary Figure S8

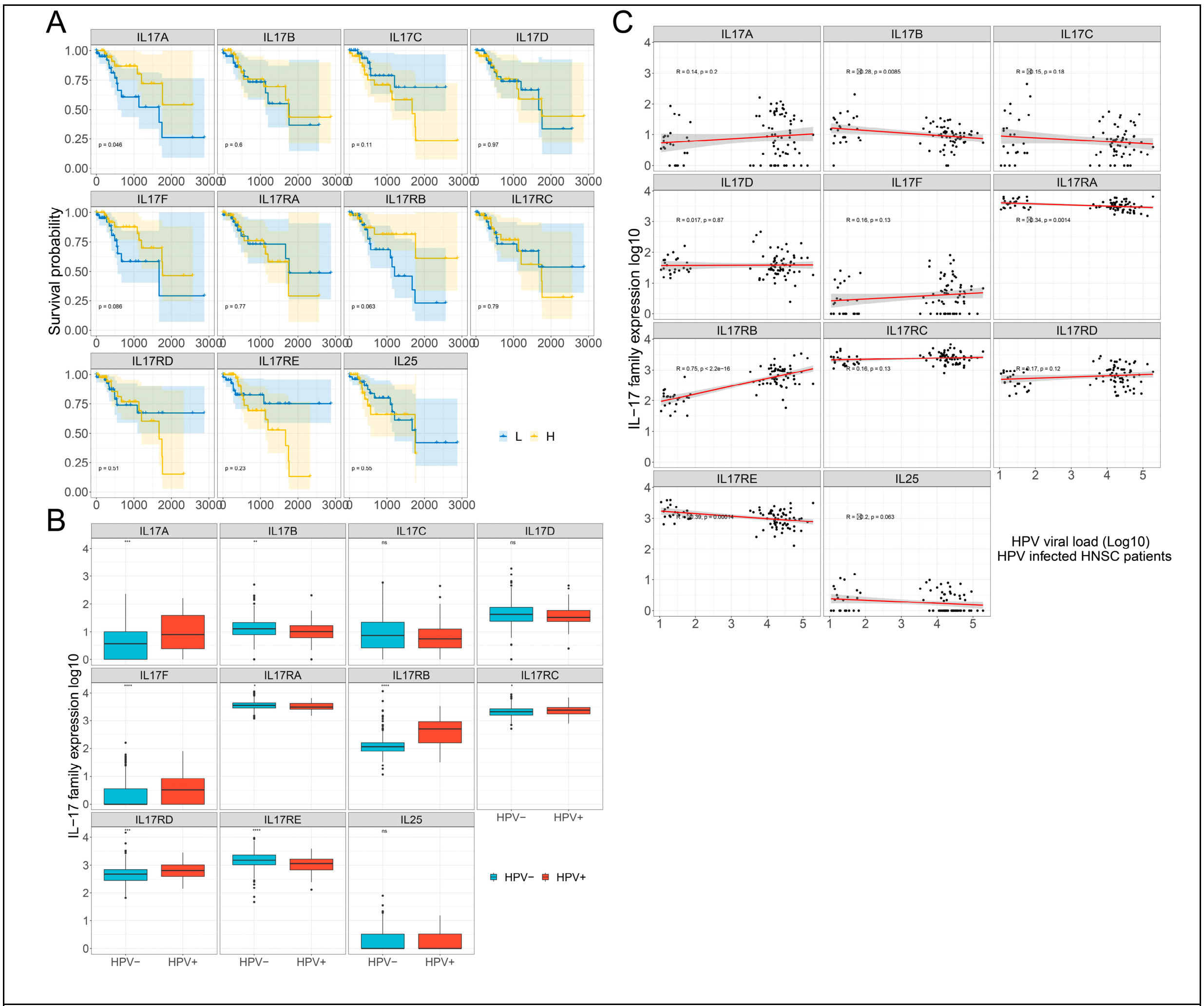

Supplementary Figure 8. Survival analysis, box plot and correlation test of IL-17 family members. **(A)** KM survival curve comparing high (yellow line) and low IL-17 family gene expression (y-axis, survival probability; x-axis, days). High tumor expression of *IL17A* and *IL17RB* was associated with improved prognosis. **(B)** Box plots comparing HNSC tumor expression of IL-17 family gene expression. *IL17A*, *IL17F*, *IL17RB*, *IL17RC* and *IL17RD* were all significantly upregulated in HPV infected HNSC patients. **(C)** Scatter plots of the correlation (red line) between HPV viral load (x-axis) and the expression of IL-17 family members. *IL17RB* expression was significantly correlated with HPV viral loads.

Supplementary Figure S9

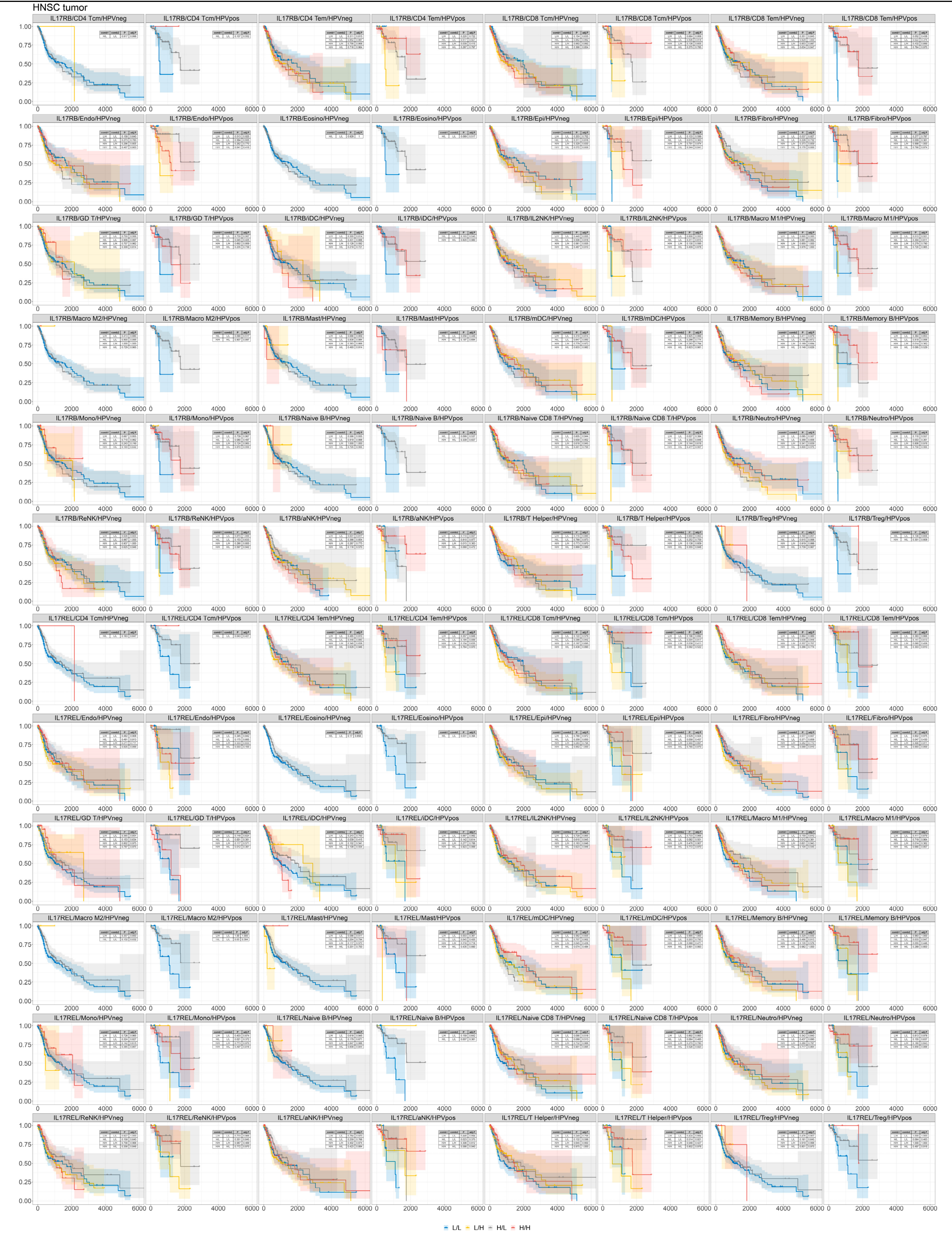

Supplementary Figure 9. Survival analysis of *IL17RB, IL17REL* and all cell TS. Combined HNSC patient survival analysis stratified for *IL17RB, IL17REL* and all cell TS expression in both HPV negative and positive patients. KM curves (y-axis, survival probability; x-axis, days) display HPV-infected or free HSNC patient survival plotted in all four combinations for each stratum (L/L, L/H, H/L, and H/H, both L and H groups were split by the median gene or cell TS expression).

Supplementary Figure S10

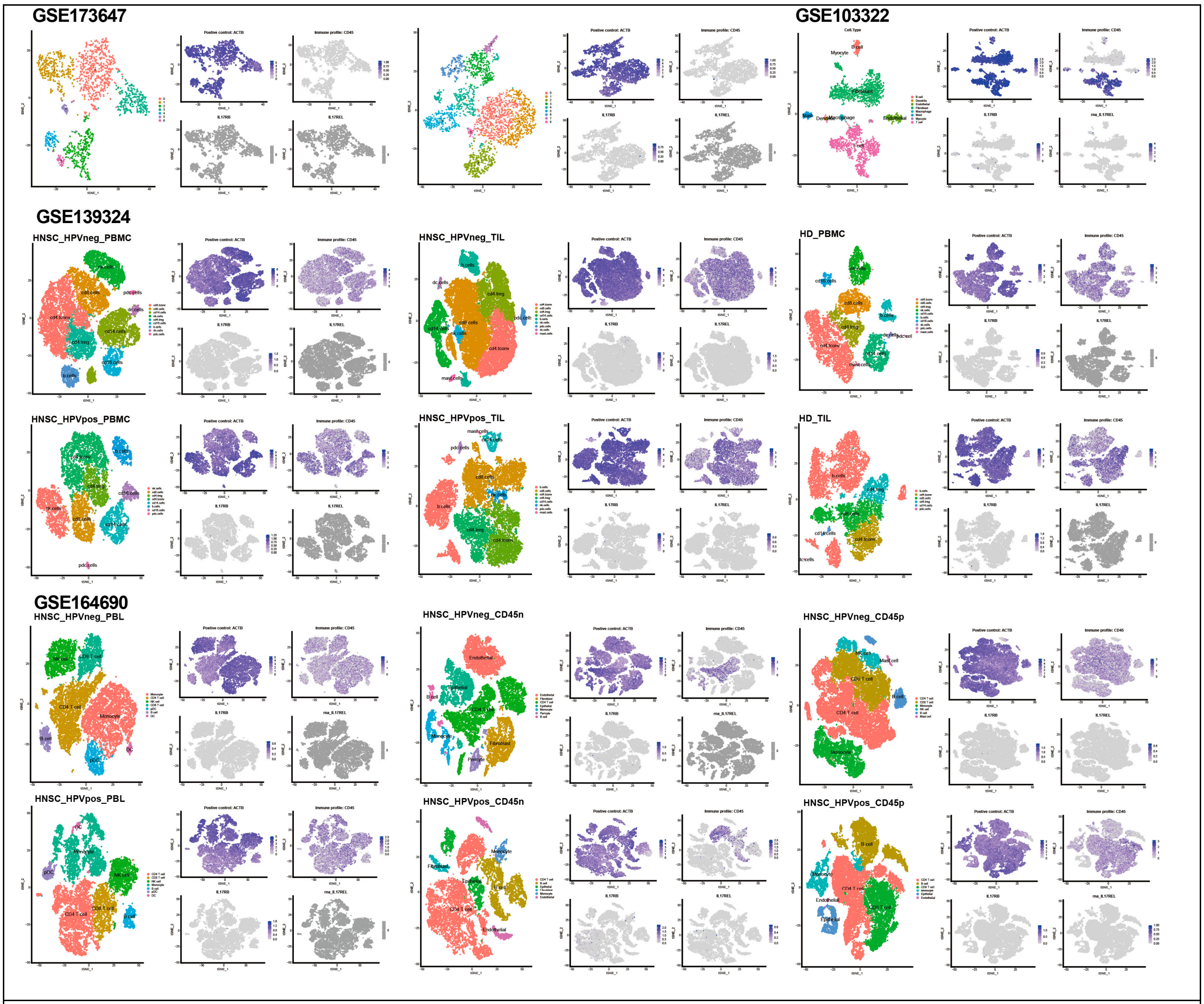

Supplementary Figure 10. scRNA-seq analysis of IL17RB and IL17REL expression cell clusters. Clustering and tSNE plots were conducted in four HNSCC scRNA-seq datasets, GSE173647, GSE103322, GSE139324 and GSE164690. The expression of *ACTB* (positive control, left top panel), *PTPRC* (CD45, immune cell marker, right top panel), *IL17RB* (left bottom panel) and *IL17REL* (right bottom panel) were highlighted for each cluster. *IL17RB* and *IL17REL* were hardly detected in all the cells in four cohorts.

Supplementary Figure S11

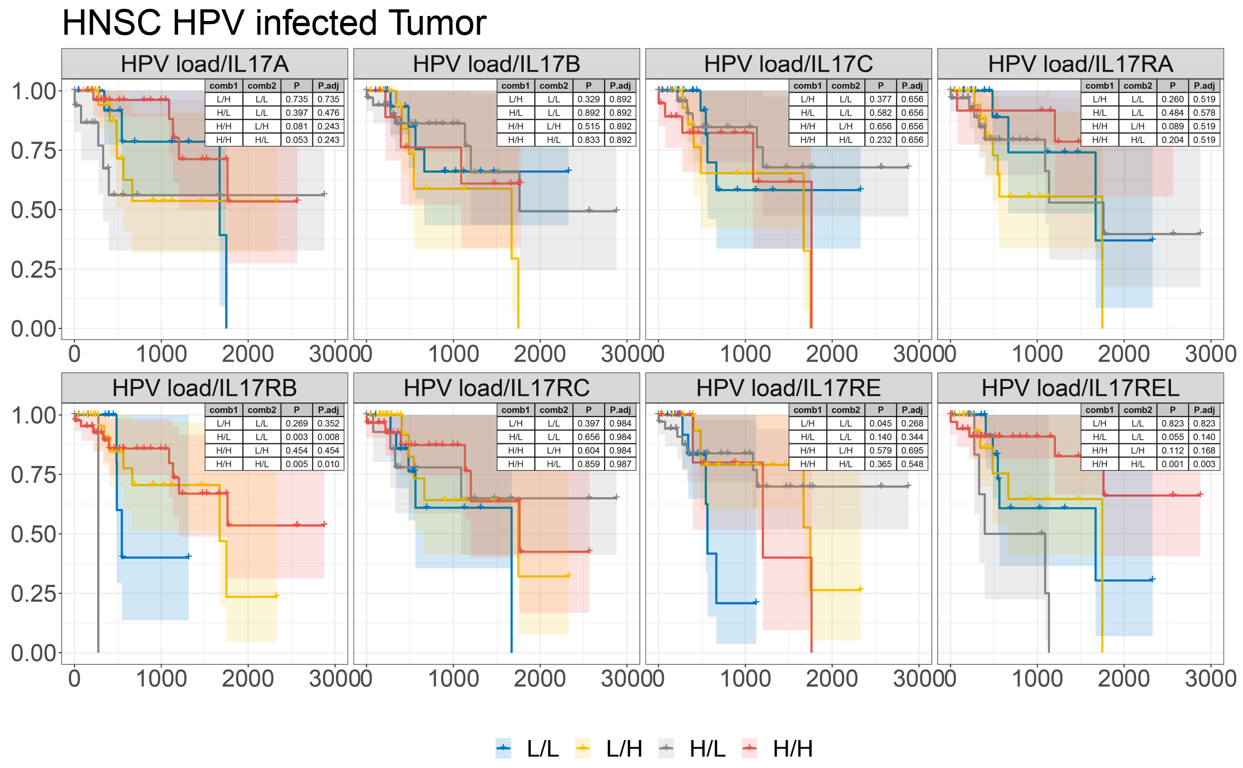

Supplementary Figure 11. Survival analysis and correlation test of IL-17 family members. Combined HNSC patient survival analysis stratified for HPV viral loads and IL-17 family members. KM curves (y-axis, survival probability; x-axis, days) display HPV-infected HSNC patient survival plotted in all four combinations for each stratum (L/L, L/H, H/L, and H/H, both L and H groups were split by the median viral load or gene expression). For patients with higher HPV viral loads, only high expression of either *IL17RB* or *IL17REL* resulted in enhanced prognosis.
