# Supplementary figures and images for: IL17RB and IL17REL Expression Are Associated with Improved Prognosis in HPV-Infected Head and Neck Squamous Cell Carcinomas
Source: Pathogens. 2023 Apr 7;12(4):572. doi: 10.3390/pathogens12040572 (PMC10143491; doi:10.3390/pathogens12040572)

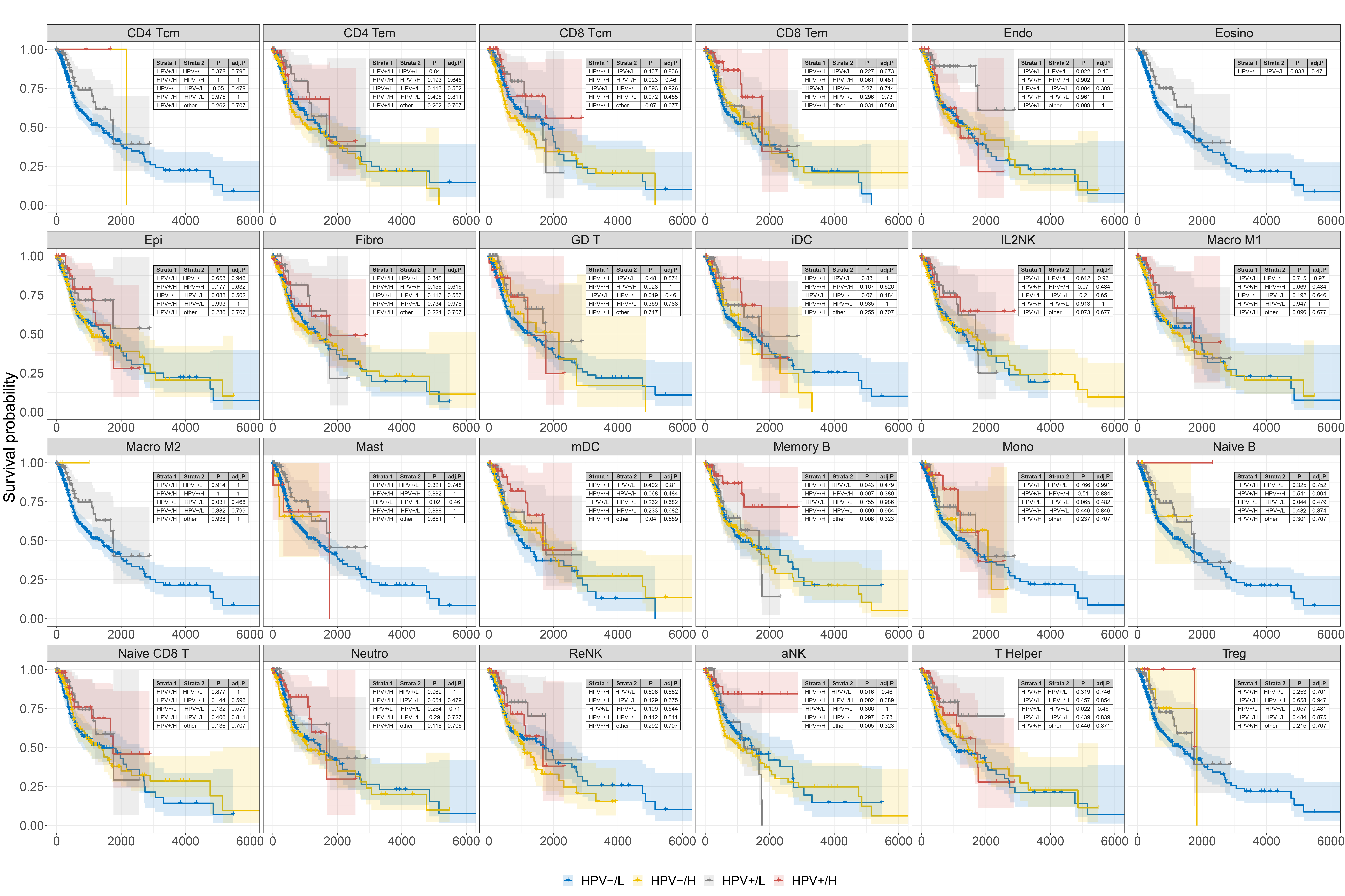

Supplement: Supplementary file 1 [file pathogens-12-00572-s001.zip › pathogens-2143972-supplementary/Supplementary Figures/Supplementary figuer 2.jpg]

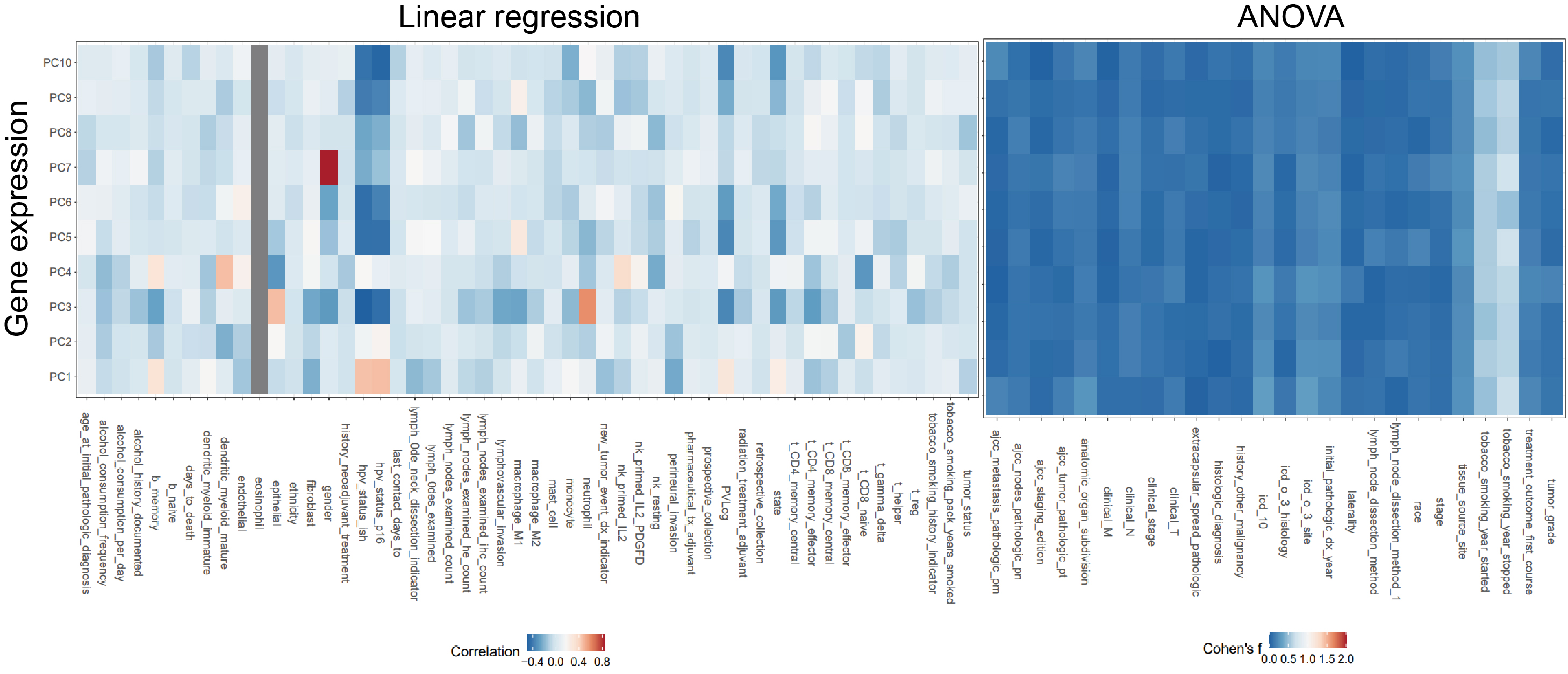

Supplement: Supplementary file 1 [file pathogens-12-00572-s001.zip › pathogens-2143972-supplementary/Supplementary Figures/Supplementary figure 1.jpg]

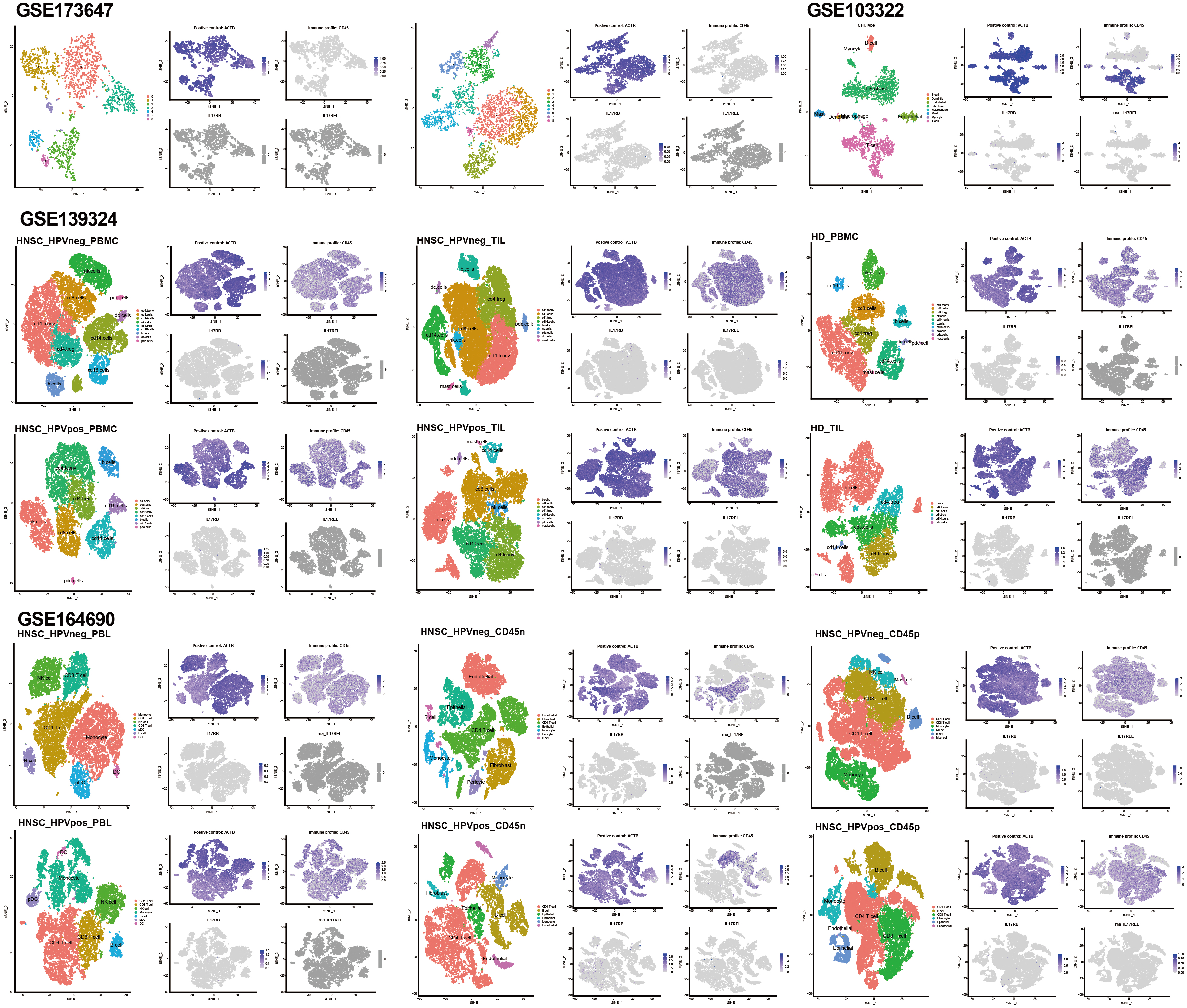

Supplement: Supplementary file 1 [file pathogens-12-00572-s001.zip › pathogens-2143972-supplementary/Supplementary Figures/Supplementary figure 10.jpg]

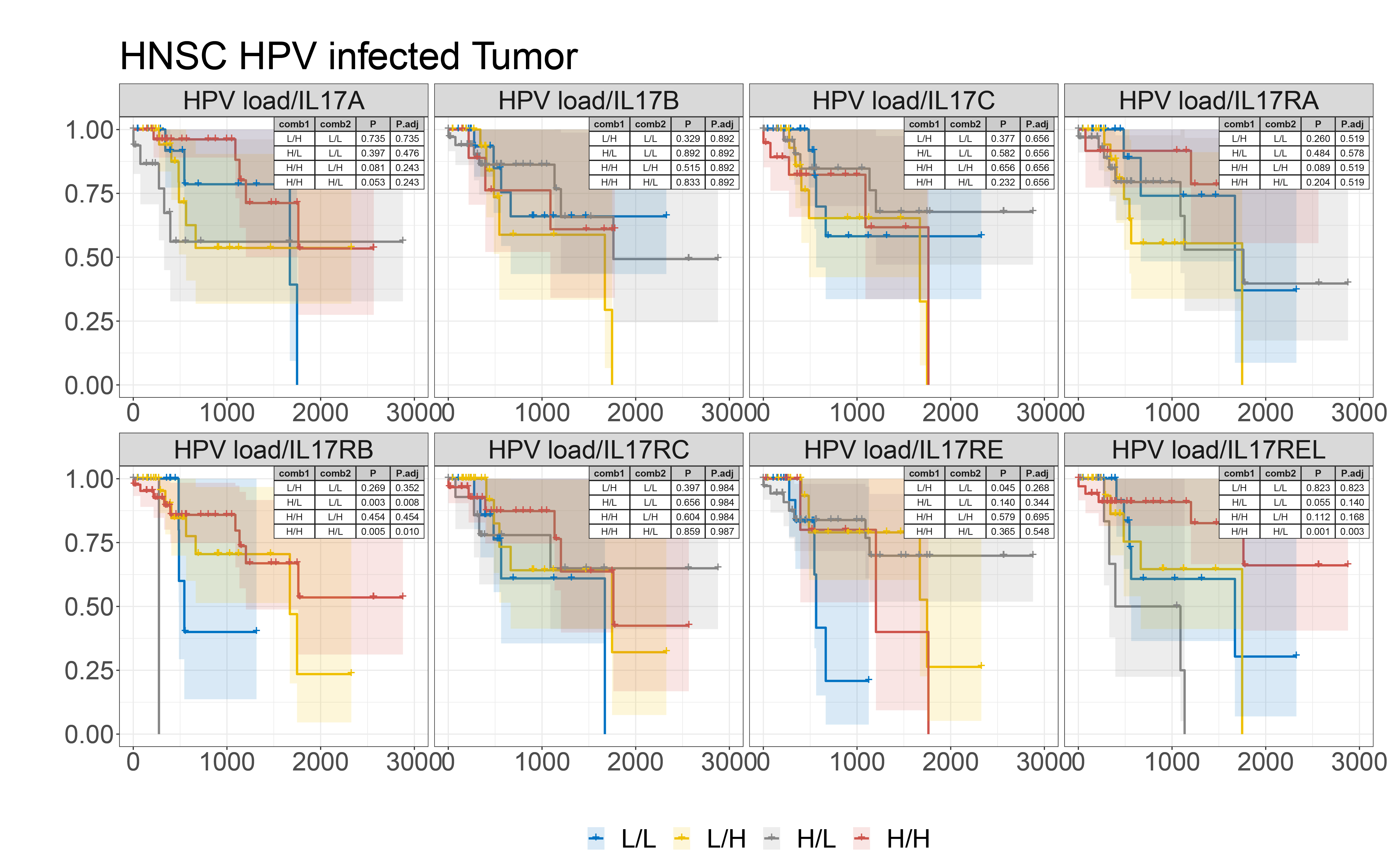

Supplement: Supplementary file 1 [file pathogens-12-00572-s001.zip › pathogens-2143972-supplementary/Supplementary Figures/Supplementary figure 11.jpg]

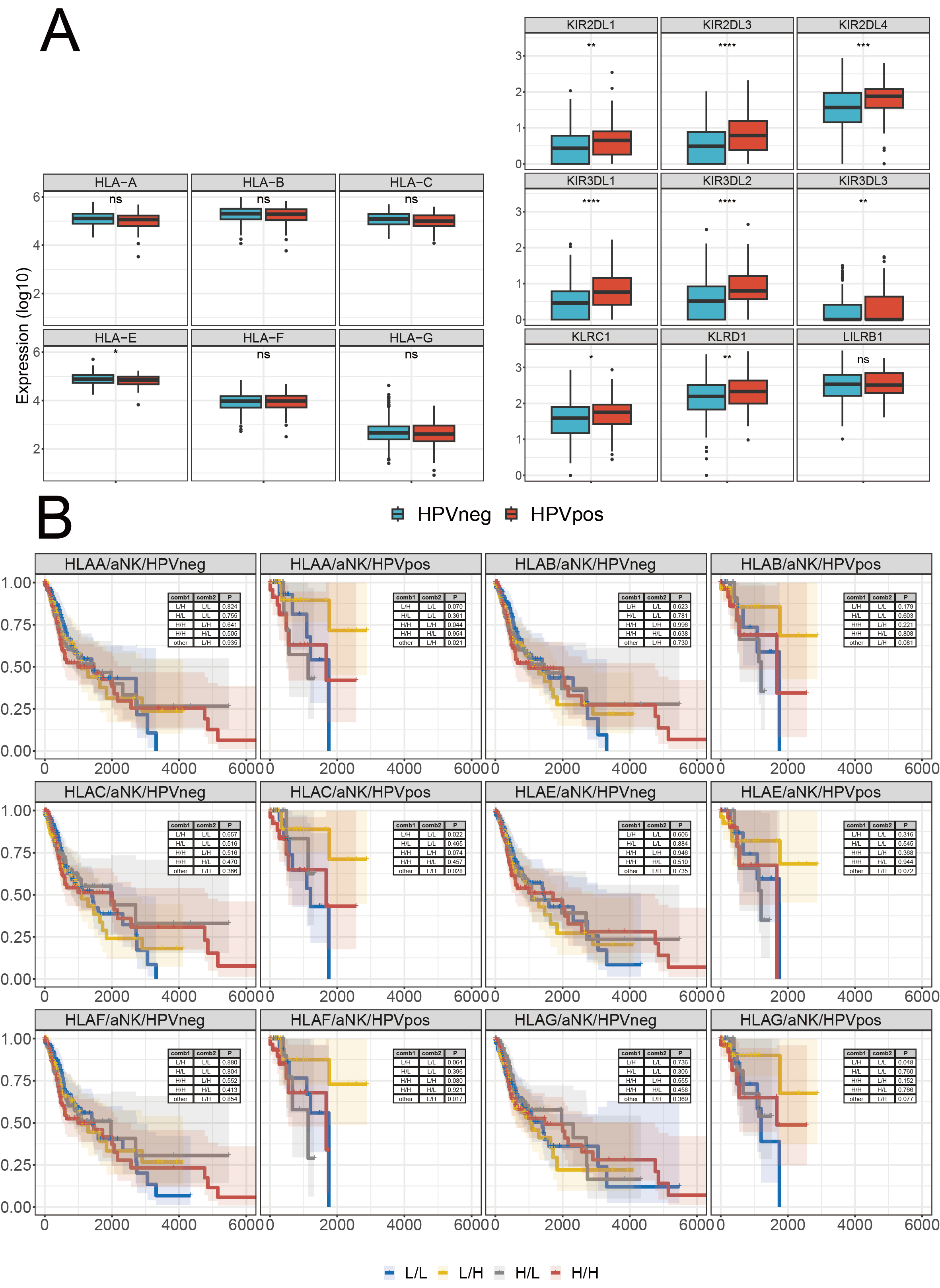

Supplement: Supplementary file 1 [file pathogens-12-00572-s001.zip › pathogens-2143972-supplementary/Supplementary Figures/Supplementary figure 3.jpg]

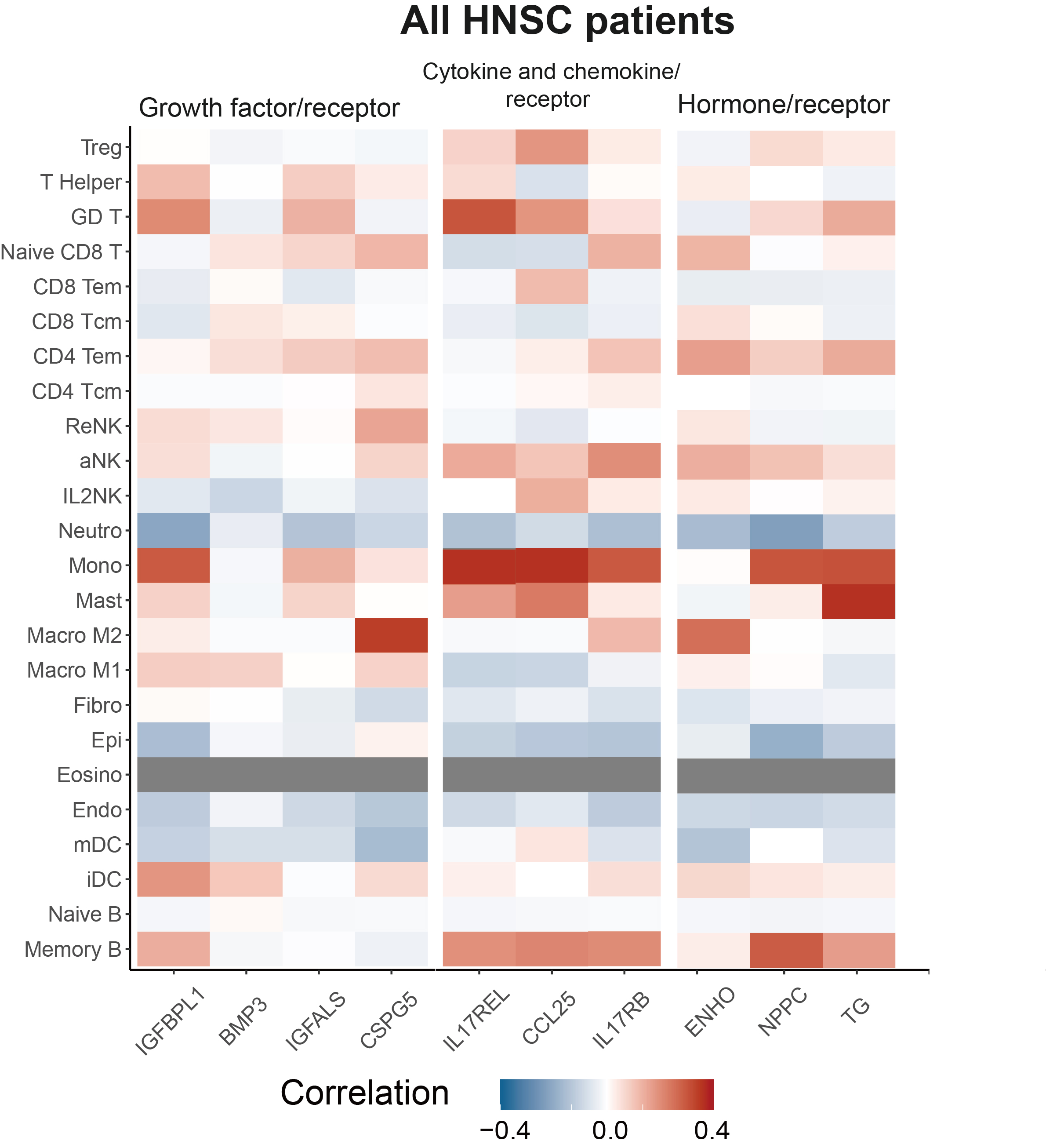

Supplement: Supplementary file 1 [file pathogens-12-00572-s001.zip › pathogens-2143972-supplementary/Supplementary Figures/Supplementary figure 5.jpg]

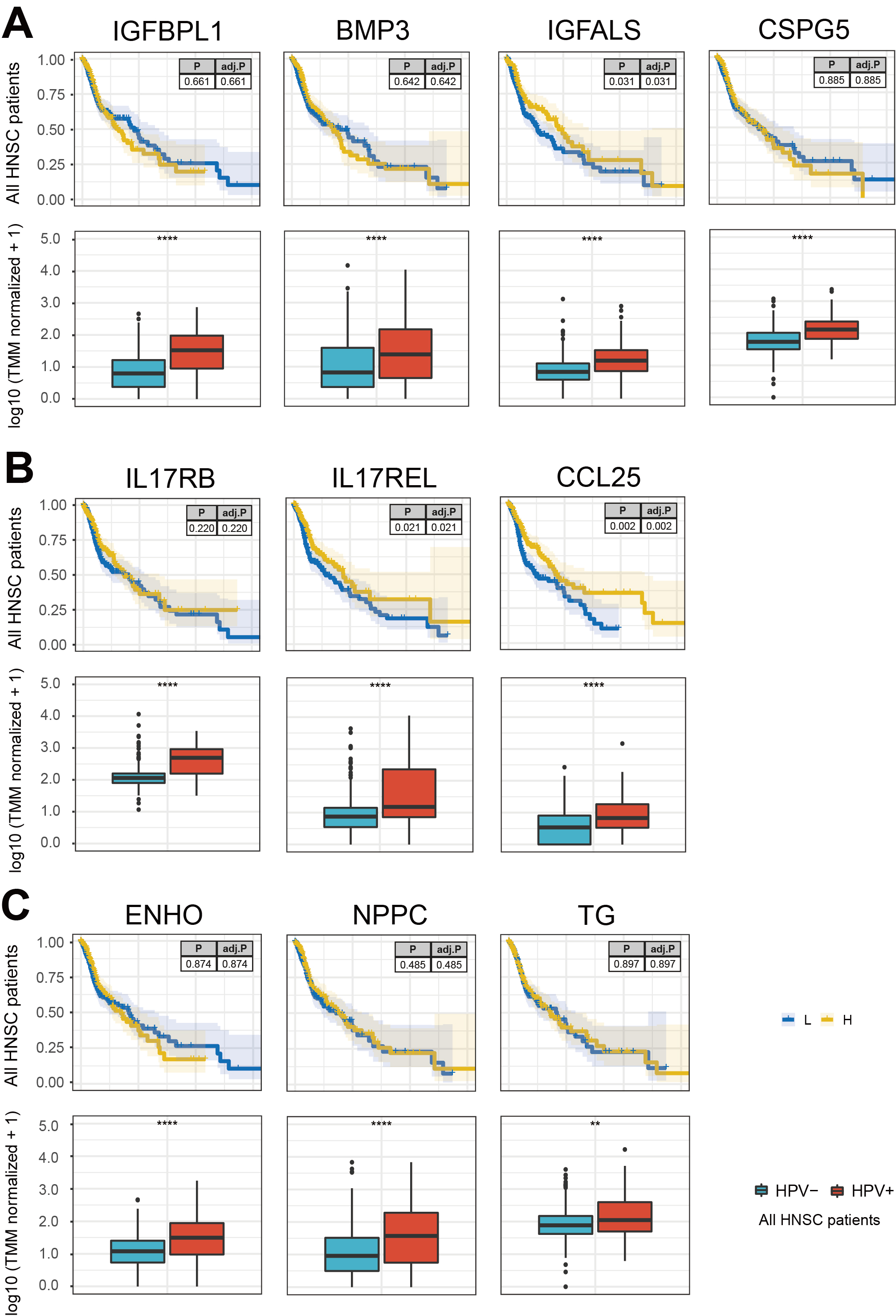

Supplement: Supplementary file 1 [file pathogens-12-00572-s001.zip › pathogens-2143972-supplementary/Supplementary Figures/Supplementary figure 6.jpg]

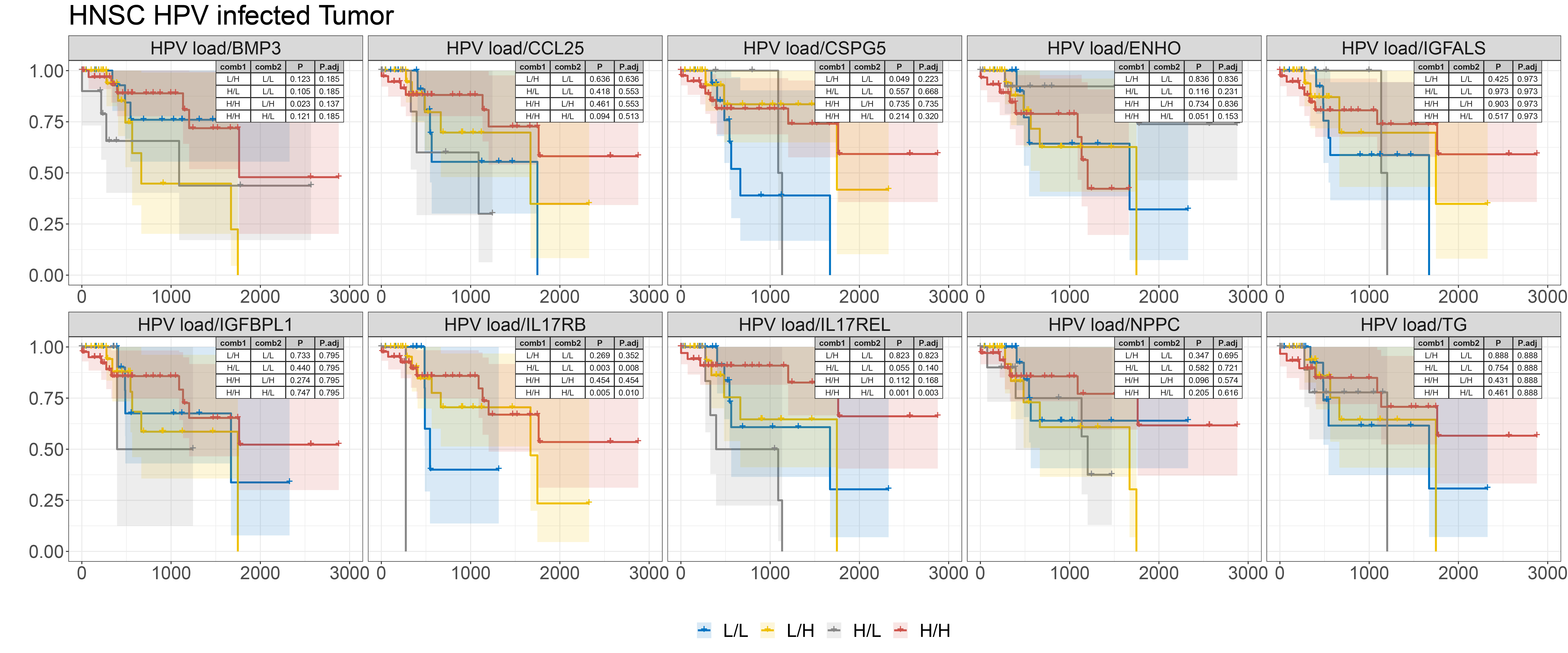

Supplement: Supplementary file 1 [file pathogens-12-00572-s001.zip › pathogens-2143972-supplementary/Supplementary Figures/Supplementary figure 7.jpg]
